# The role of oxidative stress in skeletal muscle injury and regeneration: focus on antioxidant enzymes

**DOI:** 10.1007/s10974-015-9438-9

**Published:** 2016-01-04

**Authors:** Magdalena Kozakowska, Katarzyna Pietraszek-Gremplewicz, Alicja Jozkowicz, Jozef Dulak

**Affiliations:** Department of Medical Biotechnology, Faculty of Biochemistry, Biophysics and Biotechnology, Jagiellonian University, Gronostajowa 7, 30-387 Kraków, Poland; Malopolska Centre of Biotechnology, Jagiellonian University, Kraków, Poland

**Keywords:** Reactive oxygen spices, Muscle regeneration, Superoxide dismutase, Catalase, Glutathione peroxidase, Heme oxygenase-1

## Abstract

Reactive oxygen species (ROS) are generated in skeletal muscle both during the rest and contractile activity. Myogenic cells are equipped with antioxidant enzymes, like superoxide dismutase, catalase, glutathione peroxidase, γ-glutamylcysteine synthetase and heme oxygenase-1. These enzymes not only neutralise excessive ROS, but also affect myogenic regeneration at several stages: influence post-injury inflammatory reaction, enhance viability and proliferation of muscle satellite cells and myoblasts and affect their differentiation. Finally, antioxidant enzymes regulate also processes accompanying muscle regeneration—induce angiogenesis and reduce fibrosis. Elevated ROS production was also observed in Duchenne muscular dystrophy (DMD), a disease characterised by degeneration of muscle tissue and therefore—increased rate of myogenic regeneration. Antioxidant enzymes are consequently considered as target for therapies counteracting dystrophic symptoms. In this review we present current knowledge regarding the role of oxidative stress and systems of enzymatic antioxidant defence in muscular regeneration after both acute injury and persistent muscular degeneration.

## Generation of reactive oxygen species in skeletal muscle

Reactive oxygen species (ROS), highly reactive molecules due to the presence of unpaired electron, are widely generated in eukaryotic cells as a result of incomplete, one-electron reduction of O_2_ in mitochondria. Uncoupled transfer of electron from complex I and III in the electron transport chain (ETC) leads to formation of superoxide radical (O_2_^·−^), which is a primary member of ROS (Droge 2002; Trachootham et al. 2008). During muscle contraction an increase in oxygen consumption is observed, most of which is used in ETC and reduced to H_2_O (Barbieri and Sestili 2012; Vasilaki and Jackson 2013), but as much as 5 % of O_2_ was estimated to be converted to O_2_^·−^ (Lamb and Westerblad 2011; Sakellariou et al. 2014). Therefore, initially mitochondria were regarded as a predominant and primary source of ROS in skeletal muscle tissue (Lamb and Westerblad 2011; Sakellariou et al. 2014; Vasilaki and Jackson 2013). However, more recent data reveal that the rate of electron leakage in ETC during contractile activity is in fact significantly lower, enabling only 0.15 % of total oxygen consumption to be reduced to O_2_^·−^ (Lamb and Westerblad 2011; Powers et al. 2011; Sakellariou et al. 2014; Vasilaki and Jackson 2013).

Concurrently, other metabolic pathways are implicated in ROS generation in skeletal muscle (Beckendorf and Linke 2015; Sakellariou et al. 2014), which especially during contraction, seems to be of cytosol origin (Vasilaki et al. 2006a). Among them, xanthine oxidase (XO) generates O_2_^·−^ as a byproduct of oxidation of hypoxanthine to xanthine and uric acid (Beckendorf and Linke 2015; Gomez-Cabrera et al. 2005; Sakellariou et al. 2014). O_2_^·−^ is also produced in muscles by lipoxygenases (LOX) during dioxygenation of arachidonic acid, which is released from membrane lipids due to phospholipase A2 activity (Powers et al. 2011; Sakellariou et al. 2014; Zuo et al. 2004). Finally, nicotinamide adenine dinucleotide phosphate (NADPH) oxidase (NOX) is a multicomponent enzyme system that catalyses reduction of O_2_ to O_2_^·−^ utilizing NADH or NADPH as electron donors. There are two isoforms of NOX present in skeletal muscle (NOX2, NOX4), associated with sarcoplasmatic reticulum and sarcolemma, which are currently considered as a major source of ROS in striated muscle (Beckendorf and Linke 2015; Powers et al. 2011; Sakellariou et al. 2013; Sakellariou et al. 2014).

Regardless the site of production, O_2_^·−^ has a relatively long half-life and does not react directly with proteins, carbohydrates or nucleic acids, but can serve as a substrate for generation of secondary ROS. O_2_^·−^ can be enzymatically or spontaneously dismutated and becomes, in this manner, a major cellular source of hydrogen peroxide (H_2_O_2_). H_2_O_2_ is a non-radical ROS, with a long half-life, permitting its diffusion both within a cell and across cell membranes. H_2_O_2_ is a weaker oxidising agent than its derivate—produced in a presence of reduced transition metals, the most reactive ROS, hydroxyl radical (HO^·^) (Powers et al. 2010; Powers et al. 2011; Sakellariou et al. 2014; Trachootham et al. 2008). In spite of that, H_2_O_2_ reacts with many different compounds what, in addition to its ability to diffuse, makes it an important signalling molecule (Powers et al. 2010; Powers et al. 2011; Stone and Yang 2006).

Basically, all reducing groups of main cellular macromolecules can be targeted by ROS. Lipids are most susceptible to HO^·^ that, by attacking polyunsaturated fatty acid lipid residues, generates peroxyl radical, leading to changes in properties of cellular membranes (Trachootham et al. 2008). Purine and pirimidine bases and deoxyrybose of DNA are also susceptible to HO^·^ (Trachootham et al. 2008). Finally, HO^·^ may target amino acid residues of proteins, especially lysine, arginine, histidine, proline, and threonine, causing formation of protein carbonyls. Additionally, sulfur-containing aminoacids are prone to reversible or irreversible oxidation of sulfhydryl groups (Trachootham et al. 2008). Hence, moderate ROS levels can modify signal transduction pathways in myogenic cells. ERK1/2, JNK and p38 kinases are all activated in response to ROS. Concomitantly, serine/threonine phosphatases and phosphotyrosine phosphatases are prone to oxidation-induced inactivation. These two combined ROS-promoted events activate different transcription factors and signalling pathways (Barbieri and Sestili 2012; Powers et al. 2010). Therefore any disruption in a redox control, commonly referred as oxidative stress, may lead to not only macromolecular oxidative damage, but also to potent changes in signal transduction (Bar-Shai et al. 2008; Barbieri and Sestili 2012; Kramer and Goodyear 2007; Powers et al. 2010; Powers et al. 2011). Noteworthy, among proteins which expression is dependent on ROS-induced activation of MAPK and NFκB or AP-1, one can find all major antioxidant enzymes (Barbieri and Sestili 2012).

## Major antioxidant systems in skeletal muscle

It is crucial for a cell to sustain redox homeostasis—maintain ROS below a threshold level and keep their function as a signalling molecules, while reducing their toxic effects (Barbieri and Sestili 2012). Therefore mature skeletal muscle cell as well as myogenic stem and progenitor cells are equipped with sophisticated enzymatic antioxidant systems, what renders them extremely flexible in response to changes in redox milieu (Beckendorf and Linke 2015; Powers et al. 2011).

A major class of enzymatic antioxidants, which protects against primary ROS, O_2_^·−^, is superoxide dismutase (SOD). It catalyses transformation of O_2_^·−^ to H_2_O_2_, which can be subsequently converted to H_2_O and O_2_ by other antioxidant enzymatic systems. All three existing isoforms of SOD (SOD1, 2, 3) incorporate a transition metal in their active site, to catalyse reaction of dismutation: SOD1 and SOD3 require Cu–Zn as a cofactor, whereas SOD2—Mn. SOD isoforms differ in their cellular location as well: SOD1 is located in cytosol and mitochondrial intermembrane space, SOD2 is found in mitochondrial matrix, and SOD3 acts in extracellular space (Powers et al. 2011; Trachootham et al. 2008). SOD2 was found to reduce ROS in resting muscle cells (Vasilaki et al. 2006a), SOD1 deficiency is associated with marked increase in oxidative stress in skeletal muscle (Muller et al. 2006), whereas contraction of myogenic fibre involves activation of SOD1 and SOD2 (Powers et al. 1994). Furthermore, SOD activity is elevated in oxidised myofibres, characterised by high mitochondrial volume (Powers et al. 1994).

Catalase (CAT) is a heme-dependent enzyme, localized in peroxisomes, which catalyses breakdown of H_2_O_2_ to H_2_O and O_2_ with an extremely high turnover rate—6 million H_2_O_2_ molecules/min (Powers et al. 2011; Trachootham et al. 2008). Similarly to SOD, its expression is increased in highly oxidised muscle fibres (Powers et al. 1994).

Glutathione peroxidase (GPX), a Se-dependent enzyme, reduces H_2_O_2_ or organic peroxides (ROOH) to H_2_O or alcohol (ROH), respectively. Reduced glutathione (GSH) is used as a donor of H^+^ in this reaction, and is oxidised to glutathione disulfide (GSSG) (Powers et al. 2011; Trachootham et al. 2008). GPX has lower affinity to its substrate than CAT in high concentrations of H_2_O_2_ (Pietarinen-Runtti et al. 2000; Powers et al. 2011). Like SOD, GPX is induced in muscle during its contracting and its level is elevated in highly oxidised myofibres (Powers et al. 1994), though it was also shown to be engaged in antioxidant response in resting skeletal muscle cells (Vasilaki et al. 2006a).

Expression of all three major classes of antioxidant enzymes—SOD, CAT and GPX—is elevated in a ROS-dependent manner (Kramer and Goodyear 2007; Powers et al. 2011; Trachootham et al. 2008). Specifically, two master regulators of cellular response to oxidative stress, NF-κB and AP-1 transcription factors, can bind among others to specific sites in the promoter of SOD2 (Gomez-Cabrera et al. 2005; Hollander et al. 2001; Ji et al. 2004; Lee et al. 2011; Vasilaki et al. 2006b), GPX (Vasilaki et al. 2006b) and CAT (Vasilaki et al. 2006b; Zhou et al. 2001) to induce their expression in skeletal muscle cells. ROS upregulate NF-κB through activation of IκB kinase, which phosphorylates IκB inhibitor, leading to its dissociation and, in this manner, to induction of NF-κB (Trachootham et al. 2008). On the other hand, AP-1 is a dimer composed of activating and inhibitory subunits which dimerise and interact together or with other transcription factors depending on redox milieu (Powers et al. 2011; Trachootham et al. 2008). Therefore ROS are not only the executors of muscle degeneration, but also actively trigger cellular response mechanisms, that reduce the injury (Barbieri and Sestili 2012).

Apart from SOD, CAT and GPX, which are primary antioxidant enzymes found in mammalian cells, there are also enzymes which represent second phase of antioxidant defence in skeletal muscle cells: γ-glutamylcysteine synthetase (GCS) and heme oxygenase-1 (HO-1). Although not involved in direct ROS scavenging, they are responsible for synthesis of non-enzymatic antioxidants present in skeletal muscle—GSH by GCS or biliverdin and bilirubin by HO-1 (Powers et al. 2011). GSH, apart from being a substrate for GPX, is able to reduce HO^·^ and O_2_^·−^ as well as to regenerate radical form of other non-enzymatic antioxidants (vitamin E and C) to maintain them in active, reduced state (Powers et al. 2011; Yu 1994). On the other hand, products of heme metabolism, biliverdin and its derivate bilirubin, generated by biliverdin reductase, are also reductants, though bilirubin is considered to be a better scavenger of free radicals, protecting cells from both peroxyl radicals and H_2_O_2_ (Baranano et al. 2002; Stocker 2004). In this regard, it is not surprising that GCS (Sen et al. 1992; Sen 1995) and HO-1 (Essig et al. 1997; Pilegaard et al. 2000; Saxena et al. 2010) are elevated in active skeletal muscle muscles.

NAD(P)H:quinone acceptor oxidoreductase 1 (NQO1) is an enzyme that binds FAD or FMN as a cofactor and performs two-electron reduction of quinones to their corresponding hydroquinones, using NADPH or NADH as the hydride donor. Therby, generation of one-electron reduced semiquinone and various reactive oxygen intermediates, as a result of redox cycling, is prevented. In addition to its catalytic role in reduction of quinones, NQO1 can scavenge superoxide directly, though less effectively than SOD (Dinkova-Kostova and Talalay 2010; Ma 2013). Notably, in skeletal muscles NQO1 expression elevates in response to aerobic exercise (Rodriguez-Bies et al. 2015).

Among ancillary antioxidant enzymes found in skeletal muscle, there are also the thioredoxin (TRX), glutaredoxin (GRX) and peroxiredoxin (PRX) systems (Manabe et al. 2014; Powers et al. 2011). TRX is located in cytosol and mitochondria, where it reduces disulfide proteins and concomitantly oxidises its two active cysteine residues. Disulfide bond that is created in enzymatic active centre of TRX is then reduced by TRX reductase, using electrons derived from NADPH (Arner and Holmgren 2000; Hanschmann et al. 2013). Similarly to TRX, GRX acts also in cytosol and mitochondria, where it reduces protein thiols during oxidative stress, whereas the oxidized GRX is then reduced by NADPH via glutathione reductase and GSH (Fernandes and Holmgren 2004; Hanschmann et al. 2013; Kalinina et al. 2008). Finally, PRX is a family of cysteine-dependant peroxidases, that are found in skeletal muscle cells either in mitochondrium, where they regulate ROS homeostasis (Lee et al. 2014), or in cytosol, peroxisomes and nuclei, depending on isoform (Sakellariou et al. 2014). PRX act as peroxidases, capable of reducing peroxides, H_2_O_2_ and peroxinitrates, using electrons provided by thiols such as TRX (Hanschmann et al. 2013; Kalinina et al. 2008). Since PRX molar efficacies are, however, lower than of GPX and CAT, it may play a role in regulation of H_2_O_2_ as a second messenger, in addition to its antioxidant properties (Powers et al. 2011).

Noteworthy, GCS, HO-1, NQO1, and at least some enzymes of TRX family, seem to share the same signalling pathway, governing their expression in response to ROS—their promoters contain the antioxidant repose elements (ARE) which is a binding site for Nrf2 (Chan and Kwong 2000; Hanschmann et al. 2013; Itoh et al. 1997; Ma 2013). Nrf2 is, next to NF-κB and AP-1, a third important transcription factor, which activity is regulated by redox status (Pattwell and Jackson 2004; Trachootham et al. 2008). Under normal conditions Nrf2 is localized in a cytosol, bound by inhibitor protein—Keap1. In response to oxidative stress, Keap1 is oxidized, and dissociates from Nrf2, which in turn translocates to nucleus, heterodimerizes with Maf proteins, and binds to ARE, to induce expression of ARE-dependant genes (Ma 2013; Powers et al. 2011; Trachootham et al. 2008). In this manner, transcription of both GCS (Chan and Kwong 2000; Ding et al. 2008) and HO-1 (Kang et al. 2015; Sun et al. 2015) is regulated by ROS-dependent signalling in myogenic cells.

## The complex role of oxidative stress and antioxidant enzymes during skeletal muscle regeneration after acute injury

The role of ROS and antioxidant enzymes in skeletal muscles is well confirmed not only during their physiological activity, but also during excessive exercising (Gomez-Cabrera et al. 2009), muscle fatigue (Powers et al. 2011) or aging-induced muscle wasting (Fulle et al. 2004). In this review we will focus, however, on the role of oxidative stress during skeletal muscle regeneration after injury.

Striated muscle is a stable tissue with a turnover of myonuclei only 1–2 % every week. However, this process can by tremendously enhanced after acute injury, resulting in the remarkable ability for muscle regeneration. Mechanically crushed muscle, ischemic, injected with myotoxin or destroyed by freezing, is able to successfully recover its structure and function within few weeks. To meet this, a highly coordinated, sequential, but chronically overlapping phases of muscle degeneration occur, involving myolysis and inflammation, strictly followed by regeneration, involving differentiation of muscle satellite cells (mSC) (Charge and Rudnicki 2004; Karalaki et al. 2009; Tidball 2011).

### ROS are indispensable executors and modulators of degenerative-inflammatory phase during skeletal muscle regeneration

Muscle fibre degeneration and concomitant acute inflammation begin within the first few hours post injury. Just after muscle damage, sarcolemma ruptures and myofibers undergo necrosis, what is reflected by increased plasma levels of muscle proteins (i.e. creatine kinase, myosin heavy chain). It is triggered by release of calcium from sarcoplasmic reticulum, what drives calcium-dependent proteolysis leading to tissue degeneration (Brzoska et al. 2011; Charge and Rudnicki 2004; Yin et al. 2013). Muscle necrosis activates resident mast cells, which in turn secrete cytokines (i.e. IL-1β, IL-6, TNFα) to recruit circulating inflammatory cells from the surrounding vasculature (Bentzinger et al. 2013; Brzoska et al. 2011; Pillon et al. 2013).

The first myeloid cells that invade the damaged muscle are polymorphonuclear leukocytes, mainly neutrophils, appearing already 1 h post injury and remaining elevated for at least next 48 h. They promote proinflammatory environment, necessary for clearance of cell debris, but also secrete chemokines (i.e. MIP-1α, MCP-1) to induce migration of monocytes into a site of injury. During first few days of inflammatory reaction classic Ly6C+ monocytes are predominant, expressing proinflammatory cytokines (i.e. TNFα, IL-1β) and promoting in this way further recruitment of monocytes, as well as proliferation of myogenic cells (Bentzinger et al. 2013; Saclier et al. 2013). Once monocytes invade the tissue, they begin differentiation into macrophages, which can be analogously classified into two major subtypes. Proinflammatory and phagocytic M1 macrophages (arising after exposure to TNFα, LPS or IFNγ, and secreting TNFα, IL-1β, IL-6), which are initially present in the damaged tissue, are followed by a second wave of anti-inflammatory M2 macrophages (developing after stimulation with IL-10, and releasing IL-10, TGF-β, IGF-1). Although these subpopulations are not exclusive and can be present in regenerating muscle in the same time, they have distinct functions and localization: M1 macrophages stimulate proliferation of myogenic cells and interact with them, whereas M2 reduce cytolytic damage caused by neutrophils and M1 macrophages, increase differentiation of myogenic progenitor cells and growth of myofibres, promote tissue remodelling, and can be found in close proximity to differentiating myocytes (Kharraz et al. 2013; Lemos et al. 2015; Novak and Koh 2013; Saclier et al. 2013).

At these stages of muscle regeneration ROS are massively generated, predominantly in neutrophils and M2 macrophages, what is essential for phagocytosis to occur (Barbieri and Sestili 2012; Ji 2007). Although this process is indispensable for successful muscle restoration, and ROS generated in proximity of muscle cells activate signalling pathways relevant to muscle regeneration, when oxidative stress is exacerbated it can also lead to secondary damage of previously uninjured fibres (Barbieri and Sestili 2012; Bentzinger et al. 2013; Powers et al. 2011; Tidball 2011). Noteworthy, ROS induced signalling, via activation of MAPK pathway, NFκB and AP-1, directly induces muscle protective response (Barbieri and Sestili 2012; Jackson 2008; Powers et al. 2011). Enzymatic antioxidant defence systems are induced by ROS within first days after injury: expression of SOD2 and GPX is significantly increased in injured muscle already 12 h after cardiotoxin-mediated damage, whereas induction of CAT is delayed and peaks on the 2nd day post-injury (Singh et al. 2014), followed by TRX which reaches highest level on day 7 (Vezzoli et al. 2011). What is more, murine myoblasts destroyed by contraction, induce the secretion of TRX, PRX and GRX. It suggests, that these proteins, secreted in response to oxidative stress, may act as myokines, to limit muscle injury (Manabe et al. 2014).

Not only inflammatory cells can influence level of ROS formation during muscle regeneration, but also the ROS produced in skeletal muscle can regulate post-injury inflammation. Aging-induced redox imbalance was associated with aggravated inflammatory reaction after acute damage (Ghaly and Marsh 2010). Treatment of skeletal muscle with the non-enzymatic antioxidants (Avci et al. 2012; Myburgh et al. 2012), GPX-mimicking compound (Gierer et al. 2010) and finally pharmacological stimulation of SOD2 (Togliatto et al. 2013) or HO-1 (Wilson et al. 2015) decreased inflammation in skeletal muscle after trauma. That may in turn result in reduced muscle injury (Gierer et al. 2010; Jazwa et al. 2013; Li et al. 2005; Myburgh et al. 2012). The mechanism of these effects could be possibly associated with a decrease in NF-κB activity, caused by redox imbalance. Accordingly, muscle specific impairment of NF-κB activity restricted infiltration of cardiotoxin-injected skeletal muscle with inflammatory cells (Mourkioti et al. 2006), while lack of Nrf2, accompanied by decreased expression of HO-1, TRX-1 and GCS was related to NF-κB induction, increase in TNFα and IL-1β expression and, finally, elevated inflammation and injury after myotrauma (Al-Sawaf et al. 2014; Florczyk et al. 2014; Ichihara et al. 2010).

Interestingly, opposite effects of antioxidant-deficiency on inflammation during muscle regeneration were observed in SOD3^-/-^ mice. SOD3-deficient animals had less pronounced muscle infiltration with leucocytes and macrophages (Kim et al. 2007). This might be related to the fact, that myeloid cells also express antioxidant enzymes to protect themselves from ROS-induced cell death (Pietarinen-Runtti et al. 2000). Therefore it is probable, that significant disturbance in redox homeostasis in SOD3^-/-^ mice could have led to apoptosis of inflammatory cells.

The effect of oxidative stress and antioxidant enzymes on the macrophage function and M1 and M2 polarisation during skeletal muscle regeneration has not been investigated yet. There are, however, some indications that ROS and cell defence enzymes may play a role in these processes. ROS activate expression of proinflammatory cytokines (Chandel et al. 2000) and TLR-initiated pathway (West et al. 2011) in macrophages, and thereby they are necessary for proper activity of these cells. Furthermore, ROS were found to be necessary for M1 development during diabetes progression, as in NOX-deficient mice M2 were increased along with M1 impairment (Padgett et al. 2015). Accordingly, SOD1 overexpressing animals had predominantly M2 macrophages in their lungs (He et al. 2013), whereas HO-1 induction can drive the phenotypic shift to M2 macrophages (Naito et al. 2014). Because of some contradictory findings from in vitro studies, showing that scavenge of O_2_^·−^ does not affect M1, but augments M2 development (Zhang et al. 2013), M1M2 polarisation needs to be examined also specifically in regenerating skeletal muscle.

### Antioxidant enzymes and ROS play a role during differentiation of mSC and myoblasts

To successfully complete regeneration of injured muscle, inflammatory reaction has to be strictly followed by restoration of cellular architecture in the damaged area. The essential components of the myogenic repair phase is a population of mononucleated muscle stem and progenitor cells, called muscle satellite cells (mSC). Although comprise only 2–10 % nuclei in the myofibre, they are prerequisite for muscle regeneration. mSC are located in a specific niche between sarcolemma and basal lamina. They become activated upon injury, proliferate and then differentiate into myoblasts (muscle progenitor cells), that fuse with each other to form multinucleated myotubes and, finally, mature myofibres. At each stage the mSC and myoblast functions are tightly coordinated by the sequential expression of muscle regulatory factors (MRFs—basic helix-loop-helix transcription factors: Myf5, MyoD, myogenin, Myf6) (Karalaki et al. 2009; Relaix and Zammit 2012; Yin et al. 2013).

In a steady-state condition, the quiescent mSC are arrested at the early stages of myogenic program and express Pax7 transcription factor. They are also mitotically non-active and therefore characterised by low organelle content and high fraction of heterochromatin (Montarras et al. 2013). During muscle injury, cytokines and growth factors (i.e. HGF, FGFs, IGF-1, TNFα) are released either from damaged myocytes, or from other cell types (i.e. neutrophils, macrophages, fibroblasts) (Brzoska et al. 2011; Karalaki et al. 2009). They activate mSC, which enhance the expression of Myf5 and/or MyoD, the factors that are necessary for proliferation and induction of differentiation, respectively. Activated mSC, called myoblasts, enter proliferative phase allowing the expansion of myogenic progenitors. The expression of Pax7 is downregulated, whereas cells upregulate myogenin and Myf6, and afterwards—the expression of surface proteins (i.e. β1-integrin, caveolin-3, VCAM) to proceed fusion. In the same time heterodimerization of MRFs with enhancers (i.e. MEF2) drives the expression of muscle specific genes (i.e. actins, myosins, troponins), which are essential for proper morphology and function of skeletal muscle (Dumont et al. 2015; Karalaki et al. 2009; Yin et al. 2013). Activated mSC are also able to exit cell cycle without induction of differentiation and self-renew to restore population of quiescent mSC when they delay drop in expression of Myf5 and maintain Pax7 protein, but lose MyoD. In this manner constant mSC cell number, with the potential for myogenic differentiation, is maintained even after multiple rounds of activation, ensuring life-long regenerative capacity of the muscle (Relaix and Zammit 2012; Yin et al. 2013).

The role of ROS in myogenic differentiation is complex due to the wide spectrum of ROS-induced cellular responses—low levels of ROS modulate signal transduction pathways while high ones lead to apoptosis or necrosis (Barbieri and Sestili 2012). For instance, ROS can cause inhibition of myogenic differentiation (Ardite et al. 2004; Fulle et al. 2004; Hansen et al. 2007; Sandiford et al. 2014; Sestili et al. 2009), and this effect cannot be attributed solely to increased cell death (Sestili et al. 2009). ROS can increase NF-κB activity in muscle satellite cells and myoblasts (Ardite et al. 2004; Catani et al. 2004; Zhou et al. 2001) which in turn causes reduction of MyoD level (Guttridge et al. 2000), increases cyclin D1 transcription and cell proliferation (Guttridge et al. 1999), as well as enhances expression of YY1, transcriptional repressor of myogenic genes (Wang et al. 2007). That altogether leads to inhibition of myoblasts’ differentiation (Guttridge et al. 2000). Accordingly, muscle-restricted inhibition of NF-κB (Mourkioti et al. 2006) or pharmacological suppression of its activity (Thaloor et al. 1999) enhanced regeneration after myotrauma. On the other hand, apart from being an inhibitor of myogenic differentiation, NF-κB was shown also to be required for myogenesis (Baeza-Raja and Munoz-Canoves 2004; Conejo et al. 2001). To make the situation even more complex, beside ROS-induced upregulation of NF-κB, oxidation of sulfhydryl groups in NF-κB by ROS may suppress its binding to DNA of target genes (Trachootham et al. 2008). Therefore ROS-NF-κB axis can regulate myogenesis both positively and negatively (Bakkar and Guttridge 2010; Barbieri and Sestili 2012). Similarly, ROS can also regulate in a dual manner IGF-1 signalling pathway, which enhances myoblasts differentiation and hypertrophy (Barbieri and Sestili 2012). Namely, ROS can increase phosphorylation of IGF-1 receptor (Handayaningsih et al. 2011), while decrease IGF-1 transcription (Handayaningsih et al. 2011; Sestili et al. 2009). Furthermore, expression of PGC-1α, a regulator of mitochondrial biogenesis in muscle cells (Choi et al. 2008) is inhibited by low levels of H_2_O_2_, while high levels of H_2_O_2_ enhance transcription via AMPK activation (Irrcher et al. 2009). Additionally, elevated ROS induce mitochondrial swelling and disruption (Sestili et al. 2009). Since proper function and biogenesis of mitochondria are required for successful muscle regeneration (Pawlikowska et al. 2006; Rochard et al. 2000; Wagatsuma et al. 2011), one can notice that ROS effect on myogenesis is also bifacial in the context of mitochondria.

Oxidative stress is one of the causes of apoptotic death of progenitor and mature skeletal muscle cells, since ROS trigger early events in apoptotic pathway (Barbieri and Sestili 2012). ROS alter conformation of mitochondrial pores to enhance the release of pro-apoptotic proteins (for example cytochrome c) and facilitate activity of MAPKs as well as transcription factors required for expression of both pro- and anti-apoptotic genes (Ji 2007; Primeau et al. 2002). Since accumulation of ROS in matrix is restricted by antioxidant enzymes, mainly SOD2 and GPX, they directly influence ROS-induced cell death (Ji 2007).

**Fig. 1:**
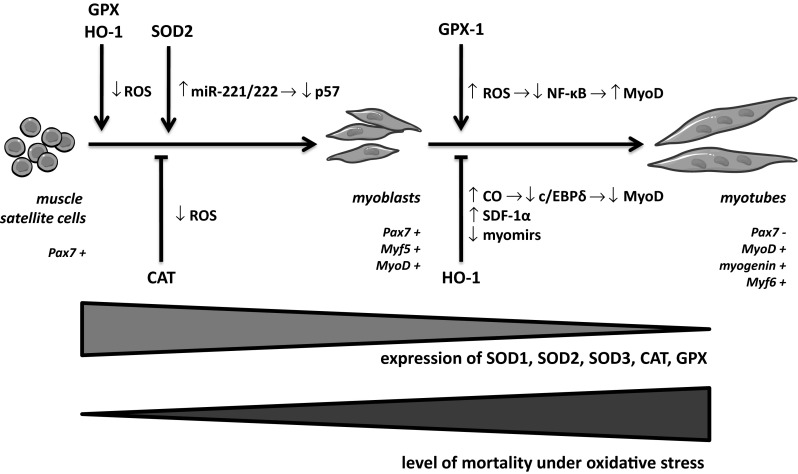
Antioxidant enzymes modulate myogenic proliferation and differentiation. SOD, GPX, HO-1 induce proliferation of mSC and myoblasts by decreasing ROS or p57. CAT may inhibit myogenic proliferation, since certain level of ROS is needed to induce it. GPX-1 was shown to augment fusion of myoblasts depending on ROS-mediated decrease in NF-κB activity. HO-1 impairs final differentiation by decreasing activity of c/EBPδ, expression of myomirs and increasing SDF-1α. Expression of SOD1, 2, 3, CAT and GPX is downregulated during progression of myogenic differentiation, what correlates reversely with the level of mortality of different myogenic populations in oxidative stress conditions. Graphic provided by http://www.servier.com/Powerpoint-image-bank was used and modified for preparation of the figure

Interestingly, it was shown that muscle progenitor cells are better protected from consequences of oxidative stress than their more mature descendants (Fig. 1). Expression of SOD1, SOD2, CAT and GPX decreases rapidly during first 24 h after the induction of differentiation in vitro (Franco et al. 1999). This lack of antioxidant defence might be somehow compensated by increased, at the same time point, the GCS activity and GSH level (Ardite et al. 2004; Ding et al. 2008). Nevertheless, after stimulation with H_2_O_2_, the quiescent mSC survive better than the activated ones (Pallafacchina et al. 2010), whereas mSC are more viable than myoblasts under the same conditions (Urish et al. 2009) and, finally, the myoblasts show reduced mortality after treatment with O_2_^·−^ donor in comparison to myotubes (Franco et al. 1999). This phenomenon correlates with increased expression of GPX-3 in quiescent mSC (Pallafacchina et al. 2010), enhanced SOD activity together with upregulated GSH level in muscle progenitors (Urish et al. 2009) as well as increased expression of SOD1, SOD2, CAT, and GPX in myoblasts (Franco et al. 1999) in comparison to their more differentiated descendants. Since high oxidative stress was shown to reduce in vivo and in vitro regenerative potential of myogenic progenitors (Ardite et al. 2004; Drowley et al. 2010; Hansen et al. 2007; Lee et al. 2006), it is not surprising that changes observed in the levels of antioxidant defence correlate with enhanced regeneration capacity of muscle progenitor cells after transplantation (Urish et al. 2009) or ischemic injury (Wilson et al. 2015).

In vivo effect of antioxidant enzymes on muscle regeneration can be attributed to the increased viability of myogenic precursors under oxidative stress. Such protective potential was shown for GPX (El et al. 2012; Lee et al. 2006; Nishida et al. 2007), SOD1 (Suzuki et al. 2004), HO-1 (Kozakowska et al. 2012; Laumonier et al. 2008; Wilson et al. 2015), or CAT (Catani et al. 2004). Accordingly, decreased level of NQO1 in skeletal muscles of Nrf2^-/-^ aged mice was correlated with enhanced apoptosis, although it must be noted, that Nrf2 deficiency was accompanied also by decreased expression of CAT, glutathione reductase and GCS (Miller et al. 2012).

Antioxidant enzymes can also affect proliferation of mSC and myoblasts. Mitotic activity is decreased in muscle precursors isolated from GPX-1^-/-^ mice (Lee et al. 2006) and can be upregulated after stimulation of SOD2 in mSC (Togliatto et al. 2013) or overexpression of HO-1 in myoblasts (Kozakowska et al. 2012). However, it must be noted that a certain level of ROS can promote cell divisions and that CAT, which expression is upregulated on the 2nd day post-injury, is able to prevent it (Sciancalepore et al. 2012; Singh et al. 2014). Furthermore, pharmacological stimulation of SOD2 renders mSC mitotically active due to downregulation of p57, which is a cyclin-dependent kinase inhibitor (Togliatto et al. 2013). Of importance, a robust proliferation is necessary to provide sufficient number of myogenic progenitors at early stages of regeneration, but it should be subsequently repressed to induce differentiation. Therefore, when proliferative phase is prolonged, it could contribute to inhibition of muscle regeneration, as observed after transplantation of HO-1 overexpressing myoblasts (Kozakowska et al. 2012).

Final maturation of skeletal muscle cells and tissue regeneration can by affected by antioxidant enzymes not only via changes induced in viability and proliferation of myogenic cells, but also by regulation of differentiation process *per se*. Specifically, increased myotube formation was observed after GPX upregulation (Hidalgo et al. 2014), whereas the reverse effects are induced in GPX-1-deficient mice (Lee et al. 2006) and under reduced GSH concentration (Ardite et al. 2004). These effects are at least partially mediated via sustained NF-κB activity, induced by GSH depletion (Ardite et al. 2004), which may induce posttranscriptional loss of mRNA for MyoD, whereas increases expression of cyclin D1 and YY1 and in this manner—suppress differentiation (Guttridge et al. 1999; Guttridge et al. 2000; Wang et al. 2007).

An increase in ROS generation observed during myogenic differentiation (Ding et al. 2008; Urish et al. 2009; Won et al. 2012) was associated with a higher expression of GCS, induced by Nrf2 (Ding, Choi et al. 2008) or upregulated transcription of PRX-2 mediated by NF-κB (Won et al. 2012). However, contradictory results were also reported. Namely, inhibition of GCS impaired myogenesis (Ding et al. 2008), while knockdown of PRX-2 enhanced differentiation of myoblasts (Won et al. 2012). This may be due to compensatory action of other antioxidant enzymes, since expressions of TRX, other members of PRX family and CAT were all upregulated in response to PRX-2 inhibition (Won et al. 2012).

Noteworthy, antioxidant enzymes can affect muscle progenitor cells also in a ROS-independent manner. Pharmacological stimulation of SOD2 activity led to increased proliferation caused by SOD2-miR-221/222-dependent repression of p57 (Togliatto et al. 2013). Overexpression HO-1 potently inhibited differentiation of myoblasts (Kozakowska et al. 2012), but this was neither mimicked by the supplementation of control cells with antioxidant products of HO-1 nor by antioxidant N-acetylcysteine. Instead, the inhibition was conveyed by HO-1-derived carbon monoxide (CO), which impaired binding of c/EBPδ transcription factor to *myod* promoter. This resulted in inhibition of MyoD expression and, consequently, in the blockage of terminal myogenic differentiation. HO-1 inhibited also generation of muscle-specific miRNA (myomirs): miR-1, miR-206, and miR-133a/b (Kozakowska et al. 2012). Noteworthy, when expression of HO-1 was temporally regulated, to be induced only during first days after ischemic muscle injury, it decreased mortality in muscle tissue, enhanced myoblast proliferation and inhibited drivers of differentiation, such as myogenin and miR-206. Switching off HO-1 at later time points improved myogenic differentiation by upregulating miR-206 and myogenin (Jazwa et al. 2013). It may be supposed that temporal overexpression of HO-1 initially promotes myoblast proliferation at early phase of regeneration, as suggested by increase in Pax7 accompanied by inhibition of MyoD and myogenin. Then switching off HO-1 may accelerate muscle differentiation by downregulating Pax7 and miR-146a, while upregulating miR-206 and myogenin (Jazwa et al. 2013). Similar results were recently reported regarding Nrf2, which was shown to induce proliferation and inhibit differentiation of mSC (Al-Sawaf et al. 2014).

### Effect of antioxidant enzymes on processes accompanying skeletal muscle regeneration: angiogenesis and fibrosis

Although angiogenesis and fibrosis are not classically regarded as components of skeletal muscle restoration, they are in fact tightly correlated events. Since the vast majority of muscle progenitor cells are localised near blood vessels, and the more vascularised muscle is, the more mSC are present, it is not surprising that regeneration of the injured muscles involves simultaneous tissue revascularisation to restore blood supply (Bentzinger et al. 2013; Yin et al. 2013). Fibrosis is defined as formation of a connective tissue scar due to prior activation of fibroblasts and fibro-adipogenic progenitors during inflammatory phase. Although fibroblasts are important components of mSC niche, as they secrete cytokines and growth factors, a balance between fibrosis and myogensis is necessary to provide optimal muscle regeneration and recovery of contractile function. Especially, that major profibrotic factor, TGF-β, impairs differentiation of mSC (Bentzinger et al. 2013; Gharaibeh et al. 2012).

The role of antioxidant enzymes in angiogenesis related to muscle regeneration was studied in a hind limb ischemia model, where disruption of oxygen supply resulted in induction of angiogenic response (Silvestre et al. 2013). In this experimental setting, SOD3 deficiency was shown to inhibit capillary formation due to increased O_2_^·−^ production (Kim et al. 2007), whereas the opposite effect was induced by SOD3 overexpression (Oshikawa et al. 2010). Accordingly, lack of GPX-1 protein inhibited blood flow restoration (Galasso et al. 2006), whereas pharmacological stimulation of SOD2 induced angiogenesis and in this manner contributed to increased rate of myogenic regeneration (Togliatto et al. 2013). It was also demonstrated that overexpression of TRX reduces oxidative stress in ischemic skeletal muscle and improves NO bioaviabilty, thereby inducing angiogenesis (Dai et al. 2009). Although the level of skeletal muscle regeneration was not estimated, one can suppose that potent changes in angiogenesis may promote also myogenesis.

However, the contrary effects of antioxidant enzymes on angiogenesis were also described. Overexpression of GRX-1 in skeletal muscle led to increased activity of NFκ-B due to removal of GSH adducts from p65 subunit. That caused the increased expression of soluble VEGF receptor (sFlt) and, in consequence, inhibited angiogenesis as well as function of post-ischemic muscle (Murdoch et al. 2014).

Finally, it is also possible, that the effects of an enzyme on angiogenesis and myogenesis are opposing. HO-1 was shown to induce angiogenic response after hind limb ischemia due to upregulation of proangiogenic growth factors: SDF-1α and VEGF (Kozakowska et al. 2012; Kozakowska et al. 2015; Lin et al. 2009; Tongers et al. 2008). Therefore, despite the inhibition of myoblast differentiation, potentially exerted also by SDF-1α (Kozakowska et al. 2012; Odemis et al. 2007), HO-1 can improve muscle regeneration after ischemia at least partially due to increased angiogenesis (Jazwa et al. 2013).

As a final point, antioxidant enzymes are believed to interfere with the progression of fibrosis in skeletal muscle. Development of fibrotic tissue during myogenic regeneration was observed in aged animals due to increased TGF-β expression (Ghaly and Marsh 2010). In aged muscles the disturbance in redox status and expression of antioxidant enzymes was also reported (Fulle et al. 2004). Apart from that, the reverse correlation between total SOD activity and progression of fibrosis in skeletal muscle was also shown (Wang et al. 2013). Although HO-1 reduces expression of TGF-β (Stachurska et al. 2013), and lack of Nrf2 causes extensive muscular fibrosis after myotrauma (Al-Sawaf et al. 2014), the potential antifibrotic effect of HO-1 in skeletal muscle regeneration was not so far examined.

## Oxidative stress in Duchenne muscular dystrophy

The muscular dystrophies comprise more than 30 genetic syndromes, which are categorized by progressive skeletal muscle wasting and degeneration. They share common histological features, including variation in myofibre size, myofibre degeneration and regeneration, and the replacement of muscle with connective tissue and fat (Manzur and Muntoni 2009). Elevated ROS level is considered to contribute to the pathology of many muscular dystrophies (Tidball and Wehling-Henricks 2007). Role of oxidative stress in the progression of Duchenne muscular dystrophy (DMD) has been widely recognised and is discussed below. Oxidative stress is also involved in progression of other dystrophies and myopathies, but it has been described in detail elsewhere (Arbogast et al. 2009; Dowling et al. 2012; Terrill et al. 2013).

DMD is a lethal X–chromosome-linked recessive muscle disease, and is the most severe of progressive muscular dystrophies. The symptoms of DMD involve a broad and not easily related set of deficiencies that includes skeletal muscle weakness, inflammation, wasting and fibrosis, increased fatigability, cardiomyopathy, lower IQ, muscle metabolic defects, and synaptic dysfunction (Emery 1989; Finsterer and Stollberger 2003; Frascarelli et al. 1988; Tidball and Wehling-Henricks 2004). Despite the broad, systemic pathophysiology of DMD, the primary cause of the disease are mutations in the dystrophin gene (Koenig et al. 1989), which results in deficiency of function of the membrane-associated protein dystrophin (Emery 2002; Hoffman et al. 1988). Some other mutations in the same gene can generate a mildly defective dystrophin protein, with a less severe disease, usually with later onset, called Becker muscular dystrophy (Emery 2002). Skeletal and cardiac myofibres lacking functional dystrophin have an increased proneness to sarcolemma damage after muscle contraction, which leads to myofibre necrosis. This results in inflammation, myogenesis and new muscle formation to regenerate the tissue (Lapidos et al. 2004; Petrof et al. 1993; Rando 2001). However, repeated cycles of damage and inflammation over months and years progress to replacement of muscle by fat and fibrous connective tissues, with severe loss of muscle function, resulting in premature death, often due to respiratory or cardiac failure (Bushby et al. 2010).

Muscle damage and weakness with dystrophin deficiency are proposed to be a consequence of damage repair cycling mediated via material fatigue injury and inflammation (Rando 2002; Spencer and Tidball 2001; Warren et al. 1993). Additionally, elevated oxidative stress has been suggested as a contributing mechanism in the muscle damage and weakness in dystrophin deficiency in humans (Haycock et al. 1996; Rodriguez and Tarnopolsky 2003) and *mdx* mice (Hauser et al. 1995; Kaczor et al. 2007; Ragusa et al. 1997). Dystrophin deficient myofibers seem to be more exposed to oxidative stress, as previously reported (Rando et al. 1998b). However, conditional expression of the Polycomb group protein Bmi1 in mSC remarkable improved the muscle function in *mdx* mice by upregulation of metallothionein 1 (low molecular weight, cysteine–rich zinc binding protein). Expression of metallothionein 1 was correlated with defence against oxidative stress-induced cellular damage, resulting in delayed muscle wasting through reduction of ROS-induced oxidative stress (Di Foggia et al. 2014).

Interactions between elevated intracellular calcium level and inflammation are suggested among the causes of the prominent oxidative stress. Augmented level of cytosolic calcium leads to increase in the concentration of mitochondrial calcium and this affects ATP synthesis. Higher oxygen consumption and enhanced electron flow through the electron transport chain during increased ATP production, elevates the ROS content in muscles (Brookes et al. 2004; Feissner et al. 2009). Moreover, membrane damage during DMD progression stimulates degranulation of muscle resident mast cells as well as activation of immune cell cascade (Han 2011; Radley and Grounds 2006). Immune cells, like neutrophils and macrophages, additionally produce ROS to promote phagocytosis. Therefore, inflammatory cells, mitochondria, NOX and decoupling of NOS are the potential sources of producers of ROS in dystrophin deficiency (Spurney et al. 2008; Whitehead et al. 2008; Williams and Allen 2007). However, NAD(P)H oxidase is proposed to be the most significant source of oxidative stress and contributor to dystrophin-associated pathology in muscles. Nox2 may be activated in response to stretch in dystrophin deficient muscles, next Nox2-dependent ROS production enhance Ca^2+^ influx and generation of reactive oxygen species in *mdx* mice. Additionally, activated Src kinase leads to further activation of Nox2 via p47phox phosphorylation (Pal et al. 2014).

## Antioxidant enzymes in DMD

The possibility that oxidative stress contributes to muscle pathology in DMD was first proposed by Binder et al. (Binder et al. 1965), who noted the similarities to muscle pathology that occurred in vitamin E deficiency, which directly increases free radicals and oxidative damage. Next, Mendell and coworkers (Mendell et al. 1971) suggested that ischemia–reperfusion muscle injury produces oxidative lesions and damage with pathological characteristics, which were argued similar to those found in patients with dystrophinopathies.

Oxidative stress can be an important regulator of DMD progression. Expression of most of the enzymes associated with antioxidant defence is augmented in DMD, as well as in *mdx* mice (Disatnik et al. 1998; Kar and Pearson 1979; Matsumura et al. 2013; Ragusa et al. 1997; Rando et al. 1998b). Increased levels of lipid and protein oxidation also have been reported in animal models of muscular dystrophy (Disatnik et al. 1998; Hauser et al. 1995) and in dystrophic muscles of patients with DMD (Haycock et al. 1996; Kar and Pearson 1979; Mechler et al. 1984), and correlated in part with the degree of histopathological alterations (Ragusa et al. 1997).

SOD1 expression is significantly increased in dystrophin deficient muscles. However, due its activity is connected with conversion of superoxide to hydrogen peroxide, which can participate in lipid peroxidation, the increase in SOD1 may have a detrimental rather than protective role in DMD. Muscular overexpression of SOD1 enhanced of lipid peroxidation in the muscles and elevated muscle cytosolic proteins in serum (Rando et al. 1998a). In turn, delivery to mice the catalytic mimetic of SOD and CAT (EUK-134), which eliminate both O_2_^·−^ and H_2_O_2_, reduced markers of oxidative stress in the *mdx* diaphragm. This was accompanied with decreased macrophages and T-cells infiltration as well as improved markers of muscle damage (Kim and Lawler 2012). The overexpression of CAT by AAV gene transfer improved functions of skeletal muscles in *mdx* mice (Selsby 2011).

One of the important antioxidant pathways is the glutathione system, essential in protection against the harmful effects of ROS and oxidative stress (Bast et al. 1991). Oxidative damage in DMD was attributed to the decreased level of GSH due to lowered activity of gamma-glutamyl cysteine ligase, the rate limiting enzyme in GSH synthesis (Renjini et al. 2012). Additionally, larger activity of GSH metabolizing enzymes, GPX and glutathione reductase, with concomitantly increased GSSG:GSH ratio indicated the acute oxidative stress in the hind limb muscles of *mdx* mice (Dudley et al. 2006).

Muscle damage, inflammation and oxidative stress can activate NF-κB through phosphorylation and release of the inhibitor protein I-κB (inhibitory κB) (Gius et al. 1999). ROS can regulate NF-κB via multiple mechanisms, and ROS induced NF-κB activation has been well described (Whitehead et al. 2006). On the other hand, NF-κB is an important mediator of redox-responsive gene expression and actively involved in the upregulation of antioxidant enzymes, such as GPX and CAT, in response to oxidative stress (Zhou et al. 2001). Moreover, activated NF-κB amplifies the release of ROS and proinflammatory proteins (such as TNF-α and IL-1β), and its activity has been shown to be higher in the diaphragm and limb muscle of *mdx* mice compared with wild-type mice (Kumar and Boriek 2003; Rajakumar et al. 2013). Glucocorticoid therapy moderated high levels of NF-κB and oxidative stress, concomitant with reduced muscle damage and enhanced functional properties (Lim et al. 2004; Messina et al. 2009). Recently inhibition of NF-κB using peptide containing the NF-κB essential modulator binding domain resulted in decreased necrosis and improved regeneration in muscles of *mdx* mice (Reay et al. 2011). Moreover IRFI-042, an inhibitor of lipid peroxidation, reduced NF-κB activation and cell necrosis (Messina et al. 2006).

Additionally, role of enzymes representing second phase of antioxidant protection has been investigated. HO-1 expression was detected to be upregulated in diaphragm of *mdx* muscles. However it was mainly localized rather in macrophages infiltrating injured muscle than in muscle fibres (Hnia et al. 2007). Reduced level of HO-1 in *Stra13*-deficient *mdx* mice resulted in accelerated muscle damage as an effect of increased oxidative stress due to ROS production (Vercherat et al. 2009). The hypothesis that HO-1 could be a potential therapeutic target for DMD, is supported by studies, where sulforaphane treatment of *mdx* mice activated in skeletal muscles the HO-1 expression in Nrf2 dependent way, what resulted in reduction of infiltration of immune cells and expression of the inflammatory cytokines (TNF-α, IL-1β and IL-6) (Sun et al. 2015).

All together this suggests, that oxidative stress may act upstream of proinflammatory signalling in *mdx* mice and antioxidant treatments could be used in therapy to avoid or postpone muscle wasting in DMD. Prevention of pathology in dystrophin deficient mice by application of different antioxidants has been recently intensively investigated and reviewed (Hnia et al. 2008; Kim et al. 2013; Kim and Lawler 2012; Messina et al. 2009; Nakae et al. 2012; Whitehead et al. 2008). The favourable effects in modulating the oxidative stress were also achieved by low-level laser therapy which decreased ROS and increased activity of antioxidant enzymes such as SOD, CAT and GPX (Leal Junior et al. 2010; Macedo et al. 2015). Additionally, the low-intensity endurance exercise has beneficial influence on skeletal muscle of *mdx* mice. Its application decreased protein carbonyls in *mdx* muscles and reduced oxidative stress markers (Kaczor et al. 2007). Furthermore, in *mdx* mice after low-intensity training the level of carbonic anhydrase 3 and SOD1 was completely restored, what may suggest that this kind of treatment may counteract oxidative stress (Fontana et al. 2015).

## Conclusions

Evidences presented here suggest, that oxidative stress is an important modulator of skeletal muscle regeneration after injury. A delicate balance between ROS production and antioxidant enzymes expression and activity plays significant role in maintaining muscle homeostasis. In the first phase of skeletal muscle regeneration ROS are crucial players in induction of removal of necrotic muscle fibres and attenuating muscle injury. Later, the antioxidant enzymes as well as ROS can also affect proliferation, differentiation and final maturation of muscle progenitor cells. Additionally, the antioxidant enzymes are considered to take part in regulation of fibrosis of skeletal muscles.

Enhanced oxidative stress is proposed to be the essential mechanism in the muscle damage and weakness in dystrophin deficiency. Upregulated expression of antioxidant enzymes may be used as a marker of the disease. Prevention of oxidative stress is deliberated as a target for therapeutic treatment in muscular dystrophies. However, because of the complexity of involvement the oxidative stress in muscle homeostasis, still many questions need further investigation to explain exact role of oxidative stress in muscle regeneration.
